# Beyond the nets: fighting malaria in the 21^st^ century

**DOI:** 10.1186/1475-2875-11-S1-P74

**Published:** 2012-10-15

**Authors:** Judith Palmer Castor, Juliette Tuakli, Bonnie MacLeod, Chytanya Kompala

**Affiliations:** 1 Child Accra Inc. - Maternal Child Health Clinic, 49 Osu Badu Street - Airport West, Accra, Ghana, West Africa; 2 New York University, School of Public Health New York City, New York 10012, USA

## Background

Malaria continues to be a major public health challenge, contributing to significant healthcare burdens and a negative impact on the economic and social development of affected countries [1]. With 20% mortality, malaria is Africa’s leading cause of death for children under the age of 5 [1,2]. In Ghana, it is 50% with approximately 7.3 million cases a year [3,4]. Between 1985 and 2003, the incidence of malaria increased from 31-8% to 44.7% [2-4]. Despite efforts at early diagnosis and treatment, insecticide treated net (ITNs) distribution, residual indoor and outdoor spraying; malaria remains a serious mortality and morbidity outcome in Ghana [5-7].

This research hypothesized that there is little understanding and acceptance amongst Ghanaians regarding the long and short term health effects in malaria vector transmission therefore fundamental prevention efforts are difficult to institutionalize. This research effort examined knowledge, attitudes and behaviors regarding malaria prevention and intervention in Greater Accra in 2010 and 2011 in a Family Practice health clinic. This work was based on the paramount belief that the effective containment of malaria needs an integrated systems approach which can only happen when there is a significant change in behaviors and belief systems [8].

## Materials and methods

Patients from a family practice health clinic in Greater Accra, Ghana were surveyed in 2010 and again in 2011. Utlizing social research methods individuals were asked questions to determine their perceived risk of becoming infected with malaria and their knowledge, attitudes and beliefs towards disease. Participants were selected based on the criteria of being patients of the clinic, residents of Ghana and being parents.

## Results

In general participant responses indicated a good understanding of malaria transmission and prevention but on average participants believed that malaria transmission was inevitable suggesting that acceptance of prevention efforts is lacking. Males perceive their risk to be slightly higher by 0.5 compared to females; Older age groups had slightly higher perceived risks, with an increase of 0.2 for every seven years of age and participant with higher levels of education perceived their risks to be lower by 0.3 per every degree earned. As age increased, the number of modes of protection increase. Participants were more open towards protection usage for children versus themselves. However, participants indicated they owned nets, thought they were effective but did not use them. Overall responses did not indicate a perceived need for systematic protective products or action planning nor was there a belief real prevention efforts by the government were taking place.

## Conclusions

Findings from this work suggest that prevention efforts at the macro levels could more effective if tied to the current individual belief systems regarding malaria. Findings from this work suggest that the perception of malaria prevention is possibly linked to the lack of consistent, systematic and in-depth education regarding the disease and how it related to personal efficacy in which to effect change in one’s own environment and with medically appropriate and safe repellent solutions.
